# Upcycling of HDPE Milk Bottles into High-Stiffness, High-HDT Composites with Pineapple Leaf Waste Materials

**DOI:** 10.3390/polym15244697

**Published:** 2023-12-13

**Authors:** Taweechai Amornsakchai, Sorn Duangsuwan

**Affiliations:** 1 Polymer Science and Technology Program, Department of Chemistry, Faculty of Science, Mahidol University, Phuttamonthon 4 Road, Salaya, Nakhon Pathom 73170, Thailand; sorn.dua@student.mahidol.edu; 2 Center of Sustainable Energy and Green Materials, Faculty of Science, Mahidol University, Phuttamonthon 4 Road, Salaya, Nakhon Pathom 73170, Thailand; 3 TEAnity Team Co., Ltd., 40/494 Soi Navamintra 111, Khet Bueng Kum, Bangkok 10230, Thailand

**Keywords:** upcycling, HDPE milk bottle, pineapple leaf waste, natural fiber, high stiffness

## Abstract

In the pursuit of sustainability and reduced dependence on new plastic materials, this study explores the upcycling potential of high-density polyethylene (HDPE) milk bottles into high-stiffness, high-heat-distortion-temperature (HDT) composites. Recycled high-density polyethylene (rHDPE) sourced from used milk bottles serves as the composite matrix, while reinforcing fillers are derived from dried pineapple leaves, comprising fibers (PALF) and non-fibrous materials (NFM). A two-roll mixer is employed to prepare rHDPE/NFM and rHDPE/PALF mixtures, facilitating filler alignment in the resulting prepreg. The prepreg is subsequently stacked and pressed into composite sheets. The introduction of PALF as a reinforcing filler significantly enhances the flexural strength and modulus of the rHDPE composite. A 20 wt.% PALF content yields a remarkable 162% increase in flexural strength and a 204% increase in modulus compared to neat rHDPE. The rHDPE/NFM composite also shows improved mechanical properties, albeit to a lesser degree than fiber reinforcement. Both composites exhibit a slight reduction in impact resistance. Notably, the addition of NFM or PALF substantially elevates HDT, raising the HDT values of the composites to approximately 84 °C and 108 °C, respectively, in contrast to the 71 °C HDT of neat rHDPE. Furthermore, the overall properties of both the composites are further enhanced by improving their compatibility through maleic anhydride-modified polyethylene (MAPE) use. Impact fracture surfaces of both composites reveal higher compatibility and clear alignment of NFM and PALF fillers, underscoring the enhanced performance and environmental friendliness of composites produced from recycled plastics reinforced with pineapple leaf waste fillers.

## 1. Introduction

In recent years, the global community has increasingly recognized the urgent challenges posed by climate change and resource depletion, leading to a need for reevaluating our consumption patterns and embracing sustainable practices. One area of particular concern is the demand for materials used in everyday life, especially in the manufacturing of containers and packaging for consumer products. These items, often made of conventional plastics, have short lifespans and contribute significantly to environmental pollution [1,2,3]. While alternative materials may appear to be a logical solution, it is important to acknowledge that plastic still plays a vital role in various industries due to its versatility and cost-effectiveness.

To address the environmental impact of plastic waste, two possible approaches exist [4]: the utilization of biodegradable materials [5,6,7] and effective recycling processes [8]. Biodegradable materials offer the advantage of natural breakdown in the environment, seemingly reducing long-term pollution. However, it is important to note that only some “biodegradable plastics” are truly biodegradable [9,10], while most require specific composting conditions [11,12,13] and can even emit methane, a potent greenhouse gas, during the process [12]. Debates surrounding the advantages of biodegradability [14] versus recycling are ongoing. Research consistently supports the notion that recycling is a more energy- and resource-efficient solution [15,16,17]. By collecting and recycling plastic products effectively, substantial amounts of energy and resources can be saved compared to the production of biodegradable alternatives.

Therefore, the most effective solution to mitigate the environmental impact of plastic waste lies in the efficient recycling and upcycling of plastic products into new, valuable materials. This approach not only reduces the reliance on virgin plastics but also offers the potential for extended use and reduced carbon emissions. By reimagining the life cycle of plastic products, waste generation can be minimized, leading to a more sustainable future.

Furthermore, it is crucial to explore the integration of natural and renewable resources into these recycled materials. Biomass, including agricultural waste and natural fibers, provides a compelling avenue for sustainable composite materials. The utilization of the biological carbon stored within these materials over their lifespan offers the prospect of significantly reducing carbon emissions without compromising modern lifestyles. In this regard, pineapple leaf waste materials demonstrate promise as a potential filler in the upcycling of plastic composites.

High-density polyethylene (HDPE) milk bottles, with their distinctive appearance, are easily identifiable in the waste plastic stream. While the recycling of these bottles has been pursued for some time [18,19], recent changes in our modern lifestyle have led to a significant increase in their quantity [20]. With the rise in coffee stalls and similar establishments, the amount of discarded HDPE milk bottles has surged. This newfound abundance has renewed interest in recycling these bottles, particularly for non-food contact applications [21,22,23]. Concerns regarding potential contamination have arisen when attempting to recycle HDPE milk bottles back into food contact applications [24]. Therefore, recycling them into non-food contact applications is a safer and more practical approach.

This study focuses on the upcycling of HDPE milk bottles by incorporating pineapple leaf materials. In contrast to many natural fibers that require cultivation, pineapple leaf waste presents a unique advantage as a reinforcing material for composite materials. Unlike fibers obtained from agricultural crops, pineapple leaf waste offers a sustainable solution without the need for dedicated cultivation resources. Additionally, studies have shown that pineapple leaf fibers possess commendable mechanical properties, making them a promising candidate for reinforcing composites [25,26]. This inherent strength, coupled with the eco-friendly aspect of utilizing waste material, positions pineapple leaf waste as a compelling choice for sustainable composite development. The pineapple leaf materials consist of pineapple leaf fibers (PALFs) and non-fibrous materials (NFMs) [27], which can be used to enhance the properties of plastic composites [28]. PALF has been shown to possess excellent reinforcing efficiency and, when implemented properly, can effectively improve the properties of both plastics [29,30] and rubbers [31,32,33]. Through a systematic investigation, our aim is to explore the effect of composition of these fillers, ranging from 0% to 20% by weight of the total composite, on mechanical and thermal properties of the composites. Although similar works exist [34,35,36,37,38,39], this study distinguishes itself by uniaxially aligning the reinforcing fibers to maximize their reinforcement efficiency.

By combining used HDPE milk bottles with pineapple leaf materials, which are abundant in Thailand and tropical countries and often discarded as waste, we seek to demonstrate the potential for transforming these materials into high-stiffness, high-heat-distortion-temperature (HDT) composites. The comprehensive characterization of these composites will provide valuable insights into their mechanical and thermal behaviors, paving the way for their application in various industries. By transforming plastic waste into functional composite materials, our aim is to contribute to the reduction in resource depletion, carbon emissions, environmental pollution, and the downgrading associated with conventional recycled plastics.

## 2. Materials and Methods

### 2.1. Materials

**Figure 1:**
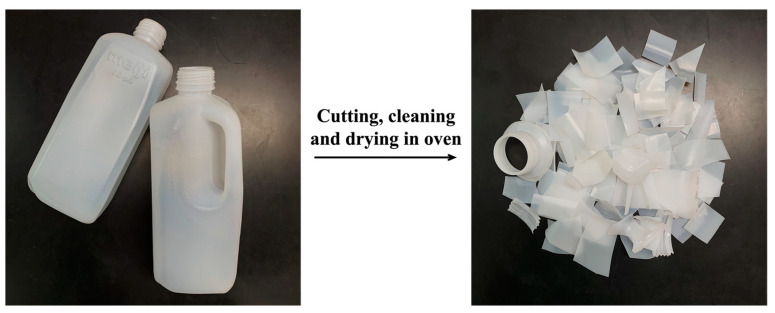
HDPE milk bottles and rHDPE obtained after cutting, cleaning and drying.

Recycled high-density polyethylene (rHDPE) from milk bottles was used in this research. The bottles were mainly 2 L milk bottles of mixed brands collected from coffee shops and stalls in the vicinity of the university. The bottles were cut and cleaned, then dried in an oven, as shown in Figure 1. Pineapple leaf materials, i.e., non-fibrous material (NFMs) and short pineapple leaf fibers (PALFs, ~6 mm in length), were prepared from fresh pineapple leaves using the procedure presented in the literature [27]. Fresh pineapple leaves were obtained from Bang Yang District, Phitsanulok Province, Thailand. The leaves were cut across their length into pieces of 6 mm long, ground with a stone grinder, and dried to yield whole ground leaf (WGL). WGL is then crushed with a high-speed blender and sieved to separate the NFMs and PALFs. The compatibilizer used was maleic anhydride-modified polyethylene (MAPE, FUSABONDTM E226), manufactured by Dow Chemical Co. Ltd. (Kingsbury Crescent, Staines, UK). The compatibilizer has a density of 0.93 g/cm^3^ and a melt flow index of 1.75 g/10 min (190 °C, 2.16 kg).

### 2.2. Composite Preparation

Before the melting mixing process, the rHDPE, NFMs, and PALFs were dried overnight in a hot air oven at 80 °C. The ratios of rHDPE and each type of filler are shown in Table 1. For selected formulations, MAPE was used as a compatibilizer at 10 wt.% of the total mass. The mixture was melt-blended on a two-roll mill (W100T, Collin GmbH, Maitenbeth, Germany) for 15 min at a speed of 48 rpm, with the front- and back-roll temperatures set at 180 °C and 100 °C, respectively.

**Figure 2:**
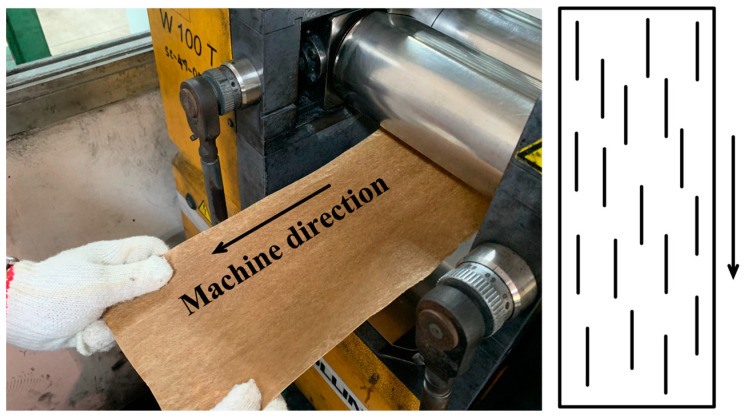
Procedure for aligning NFM and PALF on a two-roll mill during the uniaxial composite prepreg preparation. The schematic on the right shows NFM and PALF alignment as represented by black lines and the machine direction represented with the arrow.

At the end of the mixing process, the mixture was cooled slightly and pulled with slight stretching to form a uniaxial prepreg, as shown in Figure 2. The prepreg was then stacked and compressed to form 3 mm sheets at 150 °C, maintaining the prepreg aligned in the same direction. The stacked prepreg was preheated for 5 min before applying a pressure of approximately 10 MPa for 5 min and finally cooled for an additional 5 min.

### 2.3. Characterizations

#### 2.3.1. Fiber Chemical Composition

Chemical compositions of NFM and PALF were determined according to standard methods through a certified local laboratory [40,41,42,43]. Chemical composition is reported in terms of cellulose, holocellulose, acid-soluble lignin, and acid-insoluble lignin.

Surface chemical compositions of NFM and PALF were observed by Fourier-transform infrared spectroscopy in attenuated total reflectance mode (ATR-FTIR, Frontier, Perkin Elmer, Waltham, MA, USA). Spectrum were recorded with 16 scans over the range of 4000 cm^−1^ to 400 cm^−1^ with a resolution of 4 cm^−1^.

#### 2.3.2. Mechanical Properties

Flexural testing: Flexural properties of the rHDPE composite were determined using a universal testing machine (Instron 5569, Instron, High Wycombe, UK) at a crosshead speed of 5 mm/min and a load cell of 1 kN, with a support span length of 48 mm. The specimens were cut into strips of 12.7 mm width, with the long direction parallel to the machine direction. The averaged values of flexural strength and flexural modulus from five specimens were reported.

Impact testing: Impact tests were performed on a pendulum impact tester (HIT5.5P, Zwick/Roell, Ulm, Germany) in Izod mode. The impact specimens were cut from a composite sheet into strips 12.7 mm wide, with the long axis parallel to the machine direction. The specimens were notched using a Zwick/Roell manual notch cutting machine. The average values of five specimens were reported.

#### 2.3.3. Morphological Properties

The morphologies of the fillers and fracture surfaces of the composites were observed using a scanning electron microscope (SEM) (JSM-IT500, JEOL, Tokyo, Japan) with an accelerating voltage of 10 kV. Prior to observation, all samples were coated with a thin layer of platinum.

The internal structure, particularly the filler alignment within the composite sheet samples, was assessed using X-ray computed tomography (CT) (SKYSCAN 2214, Bruker Belgium, Kontich, Belgium). The source voltage was 50 kV, and the current was 150 μA, with no filter placed in front of the camera. The tungsten (W) filament served as the radiation source, and the detector was of the flat panel type. The samples were rotated 360° at a rotation step of 0.4°, with 2 averaging frames per projection. The resolution was 5 μm. The total scan time per sample was approximately 1.5 h. The images were reconstructed using NRecon reconstruction software (version 2.2.0.6) and transformed into 3D images using CTvox (version 3.3.1).

#### 2.3.4. Thermal Properties

Melting and crystallization behavior of the matrix in all composites were determined with a differential scanning calorimeter (DSC) (Q200-RCS90, TA Instruments, New Castle, DE, USA). The measurements were performed at a heating and cooling rate of 10 °C/min between 40 °C and 200 °C under nitrogen atmosphere. Melting temperature (*T*_m_), crystallization temperatures (*T*_c_) and enthalpy of fusion (Δ*H*_f_) were determined with the instrument software. The degree of crystallinity (*X*_c_) was determined using Equation (1).

(1) $$X_{c}=\left(\frac{∆H_{f}}{{∆H}_{f}^{0}\;(1-W_{f})}\right)\;\times \;100\%$$

where ${∆H}_{f}^{0}$ is the enthalpy of fusion of 100% crystalline neat HDPE, which was taken as 285.8 J/g [44], and *W*_f_ the weight fraction of the reinforcing filler in the composites.

Heat deflection temperature (HDT) was determined using a Gotech testing machine (HV-3000-P3C, Gotech, Taichung, Taiwan). The test was conducted following ASTM-D648 standards [45]. The test specimen dimensions were 120 mm × 13 mm × 3 mm. The specimens were tested under the three-point bending mode at a heating rate of 2 °C/min with a span of 100 mm, under a constant load of 0.455 MPa, and the HDT was determined as the temperature at which the specimen bends to 0.25 mm.

## 3. Results

### 3.1. Pineapple Leaf Waste Material Characteristics

The chemical compositions of NFM and PALF are presented in Table 2. PALF exhibits a significantly higher cellulose content than NFM, approximately 57.19% and 32.56%, respectively. Conversely, PALF has a lower hemicellulose and lignin content compared to NFM. These values align closely with those reported in the literature [46,47].

**Figure 3:**
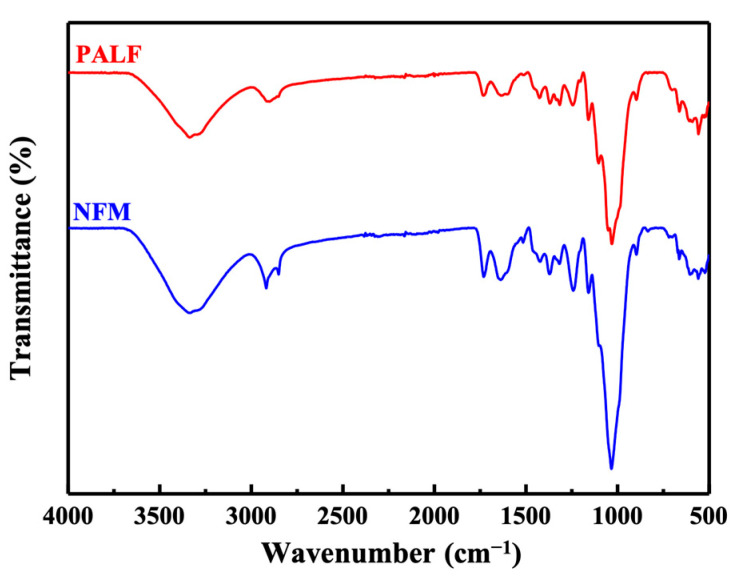
ATR-FTIR spectra of NFM and PALF.

FTIR spectra of NFM and PALF are shown in Figure 3. The FTIR spectra of both fillers did not show much difference, possibly because the two still had the same composition. The band at around 3338 cm^−1^ corresponds to the stretching vibrations of OH groups. The narrow band located at 2919 cm^−1^ is associated with C–H stretching of methyl groups (–CH_3_) in hemicellulose and cellulose [48,49]. The bands at 1735 cm^−1^ and 1244 cm^−1^ represent C=O stretching of hemicellulose and lignin and the C–O stretching of lignin, respectively [50,51]. These peaks are related to the hemicellulose and lignin components in NFM and PALF. Therefore, it can be seen that PALF has a lower peak intensity than NFM, consistent with the composition shown in Table 2. The peaks at 1424 cm^−1^ and 1369 cm^−1^ indicate CH_2_ asymmetric deformation of the cellulose and C–C stretching of the aromatic ring vibration of lignin [48,50], respectively.

**Figure 4:**
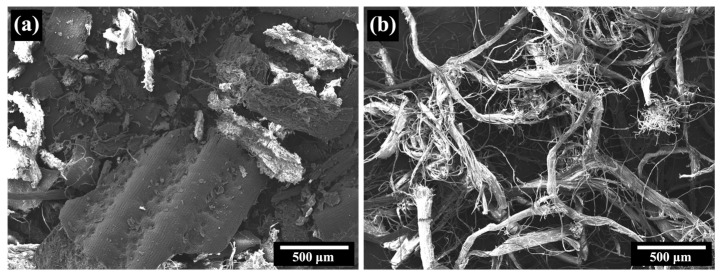
SEM micrographs of different pineapple leaf waste materials: (**a**) NFM and (**b**) PALF.

In Figure 4, SEM micrographs of NFM and PALF are displayed. NFM exhibits a particulate structure, with particles reaching sizes of up to several millimeters, whereas PALF displays a fibrous structure, with diameters ranging from a few micrometers to approximately 100 µm. It is noteworthy that most PALFs are bundles of many small fibers, which is consistent with previous research [27]. The subsequent section will delve into the effects of these filler characteristics on the mechanical properties of the composite.

### 3.2. Prepreg and Sheet Composite Characteristics

**Figure 5:**
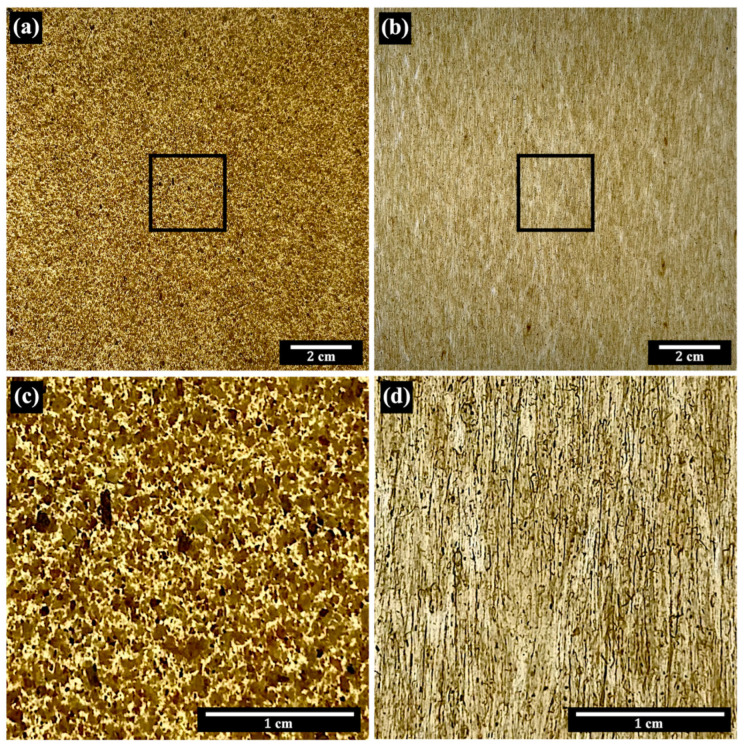
Photographs of (**a**) rHDPE/NFM and (**b**) rHDPE/PALF composite prepregs. Images (**c**) and (**d**) depict magnified views of the regions of interest indicated by squares in images (**a**) and (**b**), respectively. The machine direction is vertical.

**Figure 6:**
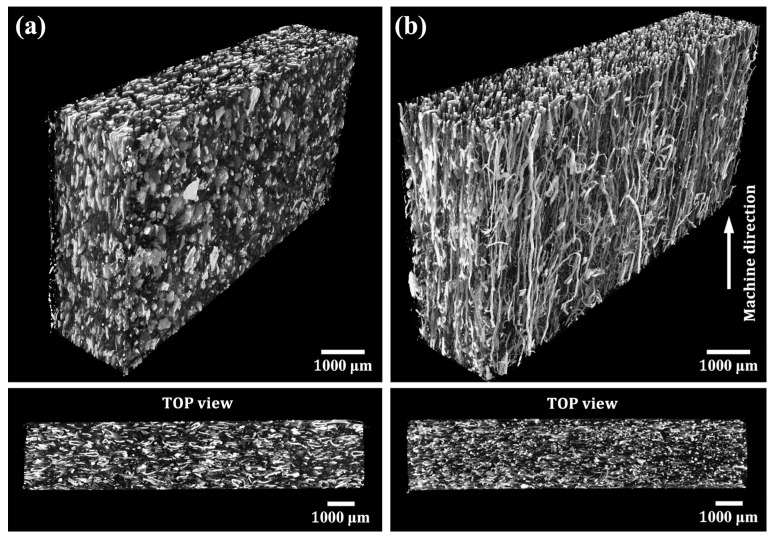
Three-dimensional X-ray computed tomography images of NFM (**a**) and PALF (**b**) composite sheets.

The appearance of the rHDPE prepreg composites, reinforced with NFM and PALF, is shown in Figure 5. PALF aligns noticeably parallel to the machine direction, while NFM alignment is less pronounced due to its particulate nature. This alignment is further evident in 3D X-ray CT images (Figure 6), where PALF fibers align uniformly in the machine direction, and NFM sheets stack clearly in alignment. The two-roll mill mixing process used in composite preparation resulted in effective filler alignment, consistent with prior research [30]. The impact of this alignment on mechanical properties will be discussed in the following section.

### 3.3. Mechanical Properties of Composites

**Figure 7:**
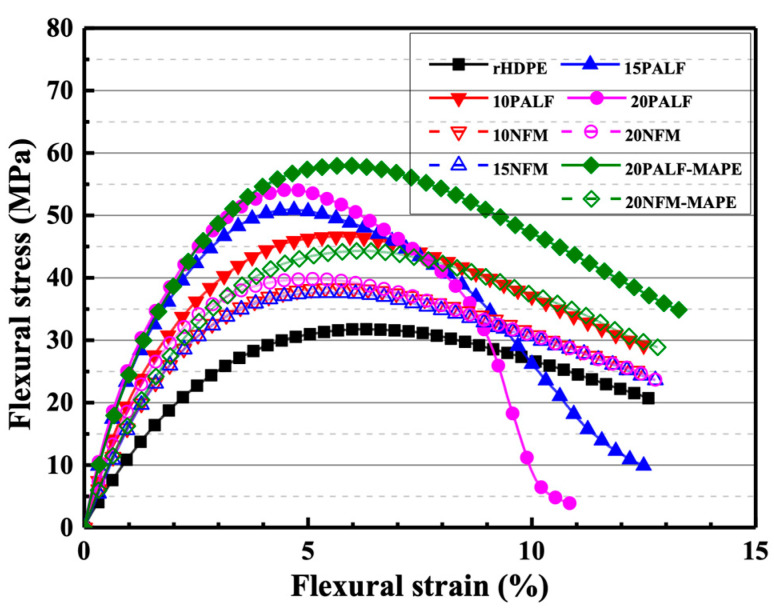
Representative flexural stress–strain curves of neat rHDPE and composite sheets containing different amounts of PALF, NFM, and adhesion promoter (MAPE). Closed symbols represent rHDPE/PALF composites, while the open symbols represent rHDPE/NFM composites.

Figure 7 displays the flexural stress–strain curves of the neat rHDPE and rHDPE composites containing different fillers. The maximum stress and slope of both composites increased with the amount of filler. Notably, the rHDPE/PALF composite exhibited higher maximum stress and slope compared to the rHDPE/NFM composite at all filler doses. Furthermore, the presence of MAPE enhanced the maximum stress of both composites, consistent with previously published results [28].

**Figure 8:**
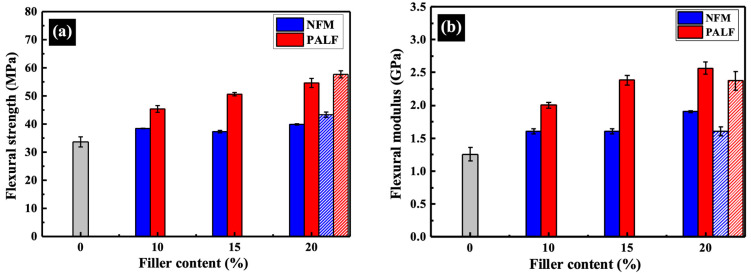
Flexural strength and modulus of rHDPE composite sheets containing different types of reinforcement: (**a**) flexural strength and (**b**) flexural modulus at 1% strain. Gray bar represents neat rHDPE. Patterned bars indicate composites with MAPE.

Flexural strength and modulus at 1% strain of the rHDPE composite with different contents of NFM and PALF are depicted in Figure 8. Both the rHDPE/NFM and rHDPE/PALF composites exhibited a flexural strength and modulus higher than those of the neat rHDPE, and these properties tended to increase as the filler content increased. Notably, PALF demonstrates better reinforcing efficiency than NFM at equal filler content, attributed to its high aspect ratio and superior mechanical properties compared to NFM [28]. In principle, according to the elastic stress transfer theory, the higher the aspect ratio, the better the fiber is able to take up stress more uniformly ([52], p. 186). Empirical findings on engineered fiber reinforced composites have revealed that the larger the fiber aspect ratio, the higher the fracture strength and fracture toughness of the composite material are ([52], p. 26).

While the flexural strength of the rHDPE/NFM composite showed little change with increasing NFM content, the modulus experienced a slight increase. However, it is worth noting that sheet-like NFM still outperforms similar materials, such as sawdust [34], or even conventional fibers [37,38,39], in terms of reinforcement performance. Part of the reason for this unexpected good performance of NFM can be attributed to the alignment of the NFM platelets during the sample preparation shown above.

The effect of MAPE on properties of the composites was tested at a dose of approximately 10 wt.%. The flexural strength of both samples increased due to better adhesion between the fillers and rHDPE [53,54]. However, the modulus decreased slightly, which could be attributed to the low molecular weight of MAPE, consistent with the literature [28].

**Figure 9:**
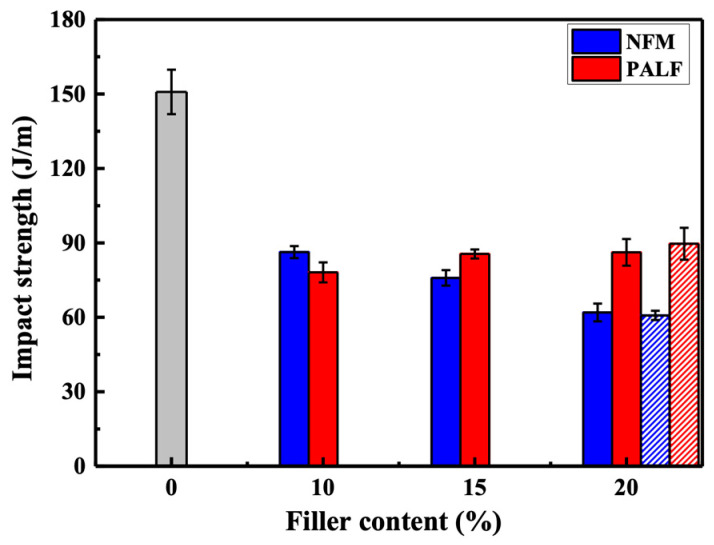
Impact properties of rHDPE composite sheets containing different types of reinforcement. Gray bar represents neat rHDPE. Patterned bars indicate composites with MAPE.

The impact strengths of all composites are depicted in Figure 9. The influence of filler types is evident as the impact strength of all samples decreased in the presence of NFM and PALF. This decrease in impact strength aligns with findings reported in other systems [36,38]. However, in the case of PALF, the impact strength tends to increase slightly as the content of PALF increases after the initial drop from that of the neat rHDPE. On the other hand, NFM continues to decrease slightly after the initial drop.

Interestingly, the addition of MAPE resulted in the enhancement of most of the overall mechanical properties of the composite. This section provides an example of the effect of MAPE on the rHDPE composites containing 20 wt.% NFM and PALF. The improvement is attributed to the greater compatibility between rHDPE and the filler, as demonstrated in several studies. However, the moduli of the samples were slightly decreased due to the lower-molecular-weight nature of MAPE compared to HDPE. The increasing compatibility between the matrix and fillers is clearly demonstrated in the fracture surfaces, which will be shown in the next section.

### 3.4. Thermal Properties

#### 3.4.1. Differential Scanning Calorimetry (DSC)

**Figure 10:**
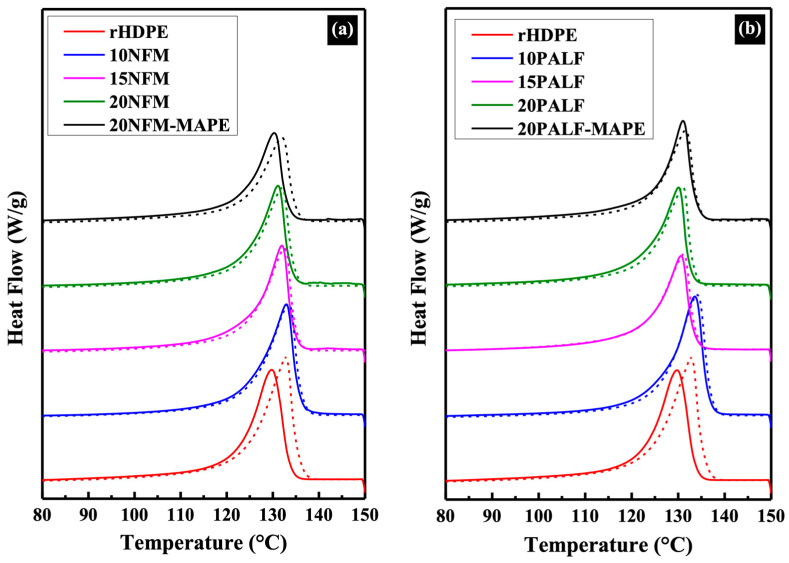
DSC thermograms of the 1st heating (solid line) and 2nd heating (dash line) cycles of the (**a**) NFM and (**b**) PALF composites.

Figure 10 presents the 1st and 2nd heating curves of rHDPE and the composites. In the case of rHDPE, variations in the thermograms can be ascribed to differences in the thermal history of the specimens. The first curve reflects cooling during the compression molding process, while the second curve corresponds to controlled cooling in the instrument. Upon the inclusion of either NFM or PALF, the influence of thermal history diminishes. The melting temperature (*T*_m_), crystallization temperature (*T*_c_), and the degree of crystallinity (*X*_c_) of rHDPE and the composites are shown in Table 3. The melting and crystallization temperature of all composite samples did not significantly differ compared to that of the neat rHDPE, with melting and crystallization temperatures in the ranges of about 130–133 °C and 118–120 °C, respectively. It is noted that the addition of fillers had little effect on the crystallization of the rHDPE. However, only a slight increase in the crystallinity of both composites was observed.

When MAPE was added, the crystallinity of the composites significantly decreased, possibly due to the low molecular weight and cluttered structure from grafted functional groups that affected the crystallization process. It has also been suggested that greater interaction (between MAPE and the filler) causes more difficult relaxation of the matrix molecules [36].

#### 3.4.2. HDT

**Figure 11:**
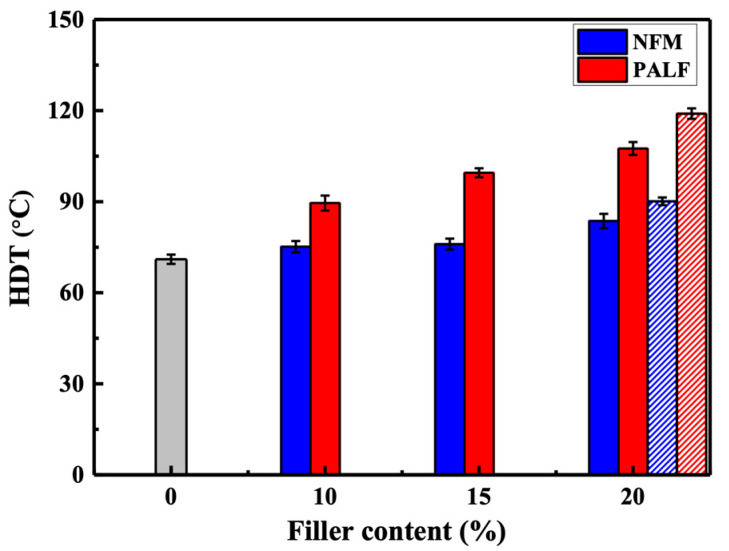
HDT of rHDPE composite sheets containing different types of reinforcement. Gray bar represents neat rHDPE. Patterned bars indicate composites with MAPE.

Figure 11 shows the HDT of neat rHDPE and composites containing different types of fillers. All composites showed a significant increase in HDT with an increasing filler content. The pattern of improvement is similar to the case of the previous flexural property. PALF offers a considerable improvement over NFM, possibly due to its higher fiber reinforcement ability than NFM, which slows down the deformation of the specimen at higher testing temperatures. The HDT of both composites (20 wt.% of fillers) was significantly enhanced upon adding MAPE.

### 3.5. Fracture Surfaces

**Figure 12:**
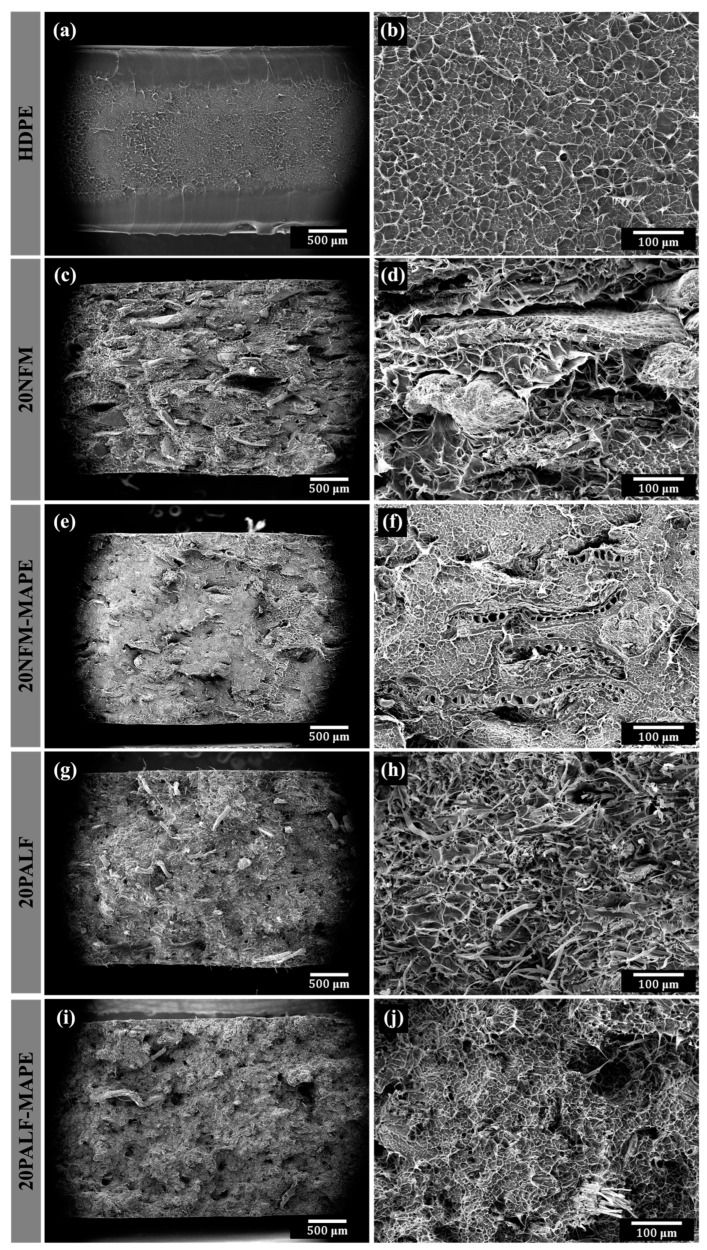
Impact fracture surfaces of rHDPE and composites containing different types of fillers at low (**left column**) and high (**right column**) magnifications. (**a**,**b**) neat rHDPE, (**c**,**d**) 20NFM, (**e**,**f**) 20NFM-MAPE, (**g**,**h**) 20PALF and (**i**,**j**) 20PALF-MAPE.

rHDPE exhibits interesting fracture surface characteristics. The edge or surface of the specimen shows a smooth fracture surface, as shown in Figure 12a,b. The surface resembles a brittle fracture. However, the central region of the fracture surface is in the form of craze fibrils, which exhibits post-impact elongation of the matrix. This is what gives rHDPE its toughness and high-impact strength.

For composites, the fracture surface clearly shows the alignment of both NFM (Figure 12c,d) and PALF (Figure 12g,h) in the same direction as the machine direction (toward the observer). This alignment is an important criterion that improves the filler’s reinforcement efficiency significantly compared to other works [39,55,56,57]. The gap between the filler and the matrix and the filler pull-out, due to poor compatibility, can be seen in both composites. However, these traits tended to decrease with the addition of MAPE. MAPE-filled composites exhibited improved compatibility between the filler and the matrix, indicated by the absence of gaps. Also, the filler shows cracks or is damaged or fractured at the cracked surface, as shown in Figure 12f,j, indicating improved adhesion. The pull-out of the filler rarely appears, especially in the PALF composite samples. This result is consistent with the previously shown mechanical properties.

## 4. Discussion and Application Example of rHDPE Composite

Previous research has indicated that recycled plastics generally exhibit lower mechanical properties. However, this study showcases a significant improvement in mechanical and thermal properties, particularly in heat distortion temperature (HDT), through the incorporation of aligned NFM and PALF reinforcements. This effect essentially upcycles the material. Notably, the level of enhancement achieved in this study surpassed that reported in comparable works [39,55,56,57]. While the previous sections provided qualitative reasoning for the high reinforcing efficiency of PALF, we will now delve into a quantitative analysis using a micromechanical model and compare our findings with published data from similar systems.

In the simplest form of the short fiber-reinforced composite model, stress transfers from the matrix to the reinforcing fibers occur via shear stress from the fibers’ ends, causing tensile stress to build up toward the center of the fibers. In effect, it can be assumed that certain parts of the fibers (both ends) do not bear the load. This model usually assumes perfect bonding between the matrix and the reinforcing fibers, with no slip occurring during deformation. Average stress can be calculated for different strains of the composite, allowing the modulus of a uniaxial short fiber composite to be determined. Several micromechanical models have been developed to predict the mechanical properties of short-fiber composites [58,59]. However, these models require numerous parameters for precise predictions, such as the mechanical properties of each individual component, shape parameters, size and length distribution, and orientation distribution of the reinforcing fibers, etc. Therefore, we prefer to use a simple model to illustrate the concept, namely the shear lag model originally proposed by Cox [60]. It can be expressed in a form resembling the modified rule of mixture, as follows.

(2) $$E_{c}=\eta_{l}V_{f}E_{f}+\left(1-V_{f}\right)E_{m}$$

where *E_c_* is the elastic modulus of the composite, *E_m_* is the modulus of the matrix, *E_f_* is the modulus of the fiber, *V_f_* is the fiber volume fraction, and *η_l_* is the shear lag factor or modulus adjustment variable for the short fiber, which includes parameters such as the shape, orientation, and arrangement of the fiber.

The fiber contribution term to composite modulus in Equation (1) may be visualized in two ways, i.e., an apparent fiber volume fraction, which is product of *η_l_V_f_* [33] or an apparent fiber modulus, the product of *η_l_E_f_* of the reinforcing short-fiber composite that is equivalent to its continuous fiber counterpart [61]. For simplicity and comparison, the latter will be considered first.

**Figure 13:**
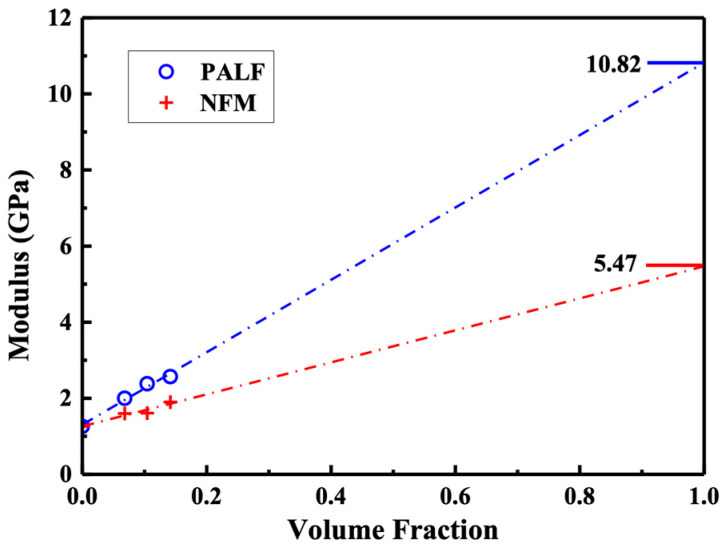
Predictions of apparent reinforcement modulus of PALF and NFM in rHDPE. Dotted line are linear fitted of the experimental results of PALF and NFM systems.

The composite modulus in Figure 8 is now plotted against the reinforcement volume fraction in Figure 13. Apparent moduli for PALF and NFM were calculated by linear curve fitting and the value extrapolated to reinforcement volume fraction of unity to 10.82 and 5.47 GPa, respectively. The apparent modulus for PALF obtained here is slightly less than 11.70 for Abaca fiber in rHDPE system containing MAPE at 8 wt.% [61]. However, it should be noted that the rHDPE/Abaca fiber samples were prepared by injection molding, in which there could be significant contribution from matrix orientation. Direct comparison of the composite modulus in Table 4 with similar published systems clearly displays the high value of both modulus and strength obtained in our study.

We will now consider the reinforcing efficiency of the fiber. By substituting the fiber modulus with a value of 20 GPa [65], we calculated a reinforcing efficiency of approximately 0.60. This value, although relatively high, becomes even more remarkable when considering the exceptionally high aspect ratio of the fibers, potentially exceeding 1000 (with a length of 6 mm and a diameter of 3–5 µm). In theory, this aspect ratio should result in a reinforcing efficiency as high as 0.8. However, the observed efficiency was lower due to incomplete fiber stretching and imperfect fiber alignment (cf. Figure 6).

The addition of MAPE did not significantly improve the modulus of the composite, which contradicts the findings in many literature sources [38,61,64]. Surprisingly, the composite exhibited a slightly lower modulus than its counterpart without MAPE. This unexpected result could be attributed to the lower molecular weight and reduced crystallinity of MAPE. Although one might anticipate enhanced interfacial adhesion between the fiber and the matrix, the layer of the matrix adjacent to the fiber became softer, leading to a decreased shear modulus and slightly less effective stress transfer to the fiber. Notably, the impact of MAPE addition became apparent only at larger deformations. This suggests an increased interfacial adhesion, causing the peak stress at which fibers could still bear the load to shift from approximately 4.5% to about 6% strain. The improved interfacial adhesion also manifested itself in the morphology of the impact-fractured surfaces (Figure 12). Although the impact strength was not affected by the addition of MAPE, fiber breakage appeared to dominate as compared to fiber pull-out without MAPE. This observation agrees well with previous published research [66] and indicates a more secure bond between the fibers and the matrix, enhancing the overall structural integrity of the composite.

The addition of MAPE significantly influenced the Heat Distortion Temperature (HDT) of the composite compared to the sample without MAPE. This effect can be attributed to the enhanced interfacial adhesion between the fiber and the matrix. HDT, measured as the temperature at which the specimen deforms under load to approximately 0.25 mm exhibited notable improvement. This amount of deflection is equivalent to a strain of only about 0.2%. Since no change in modulus (which was measured at 1% strain) was observed for the effect of MAPE, it may be postulated that the robust interfacial adhesion, resulting from polar–polar interactions between MAPE and PALF, could mitigate the time-dependent creep during the measurement, leading to a higher HDT. However, this aspect requires further in-depth investigation.

**Figure 14:**
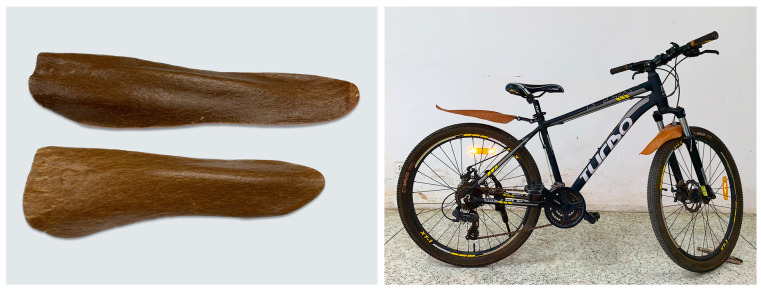
Bicycle mudguard made from rHDPE/PALF composite.

Considering the improvement obtained above, an example of a potential application for this material could be automotive or bicycle parts, where weight reduction is desirable. These products require materials with high mechanical properties and resistance to deformation under normal or high-temperature conditions. Figure 14 shows an example of a bicycle mudguard made using the composite prepreg molding technique and simple thermoforming. Because the composite material has greater stiffness and strength than conventional materials, parts can be made thinner and therefore lighter. While we were unable to demonstrate the actual weight advantage due to limited tooling, we anticipate that it would be appreciable. Therefore, this composite material could be a viable replacement for traditional materials. These results demonstrate the potential opportunities for and expansion of the use of recycled plastics. This upcycled route offers not only low-carbon-emitting products but also the opportunity to sequester carbon in the products.

## 5. Conclusions

This study demonstrates the successful upcycling of high-density polyethylene (HDPE) milk bottles through the incorporation of pineapple leaf waste materials as reinforcements. The aligned non-fibrous material (NFM) and short pineapple leaf fibers (PALFs) significantly improved the mechanical and thermal properties of the composites. PALF exhibited superior reinforcing efficiency over NFM, and the addition of a compatibilizer further enhanced the composite’s overall performance. The improved properties of the rHDPE composites open up possibilities for light weight and high-performance applications, such as bicycle, motorcycle, or automotive components, offering weight reduction without compromising mechanical integrity. This research contributes to the development of sustainable solutions by effectively utilizing recycled plastics and reducing environmental impact.

## Figures and Tables

**Table 1:** Formulation of the rHDPE composites.

Sample Code	rHDPE (wt.%)	Filler (wt.%)		MAPE (wt.%)
		NFM	PALF	
rHDPE	100	-	-	-
10NFM	90	10	-	-
15NFM	85	15	-	-
20NFM	80	20	-	-
20NFM-MAPE	70	20	-	10
10PALF	90	-	10	-
15PALF	85	-	15	-
20PALF	80	-	20	-
20PALF-MAPE	70	-	20	10

**Table 2:** Chemical composition of pineapple leaf waste materials.

Sample	Cellulose (%)	Holocellulose (%)	Lignin (%)	
			Acid Soluble	Acid Insoluble
NFM	32.56	72.62	3.04	18.50
PALF	57.19	85.49	2.61	7.82

**Table 3:** Thermal properties of rHDPE composites.

Sample	Content (%)	First Heating	Cooling	Second Heating	
		*T*_m_ (°C)	*T*_c_ (°C)	*T*_m_ (°C)	*X*_c_ (%)
rHDPE	0	129.8	119.6	132.8	73.9
NFM	10	133.0	119.6	133.5	78.2
	15	132.0	119.3	132.6	76.6
	20	131.2	119.1	132.1	75.8
	20 *	130.3	119.4	131.9	69.9
PALF	10	133.5	120.0	134.2	79.4
	15	130.6	118.3	131.1	75.6
	20	130.1	118.3	131.0	75.3
	20 *	131.1	119.2	131.5	70.5

* indicate composites with MAPE.

**Table 4:** Mechanical properties of rHDPE composites reinforced with different types of reinforcement. Data from the literature were estimated from graphs therein. The numbers in brackets are that modified with maleic anhydride-grafted polyethylene (MAPE) at 8% (Abaca) and 10% (PALF and NFM).

Fiber	Preparation Method (Mixing/Molding)	Flexural Strength			Flexural Modulus			Ref.
		Matrix (MPa)	Composite (MPa)	Increment (%)	Matrix (MPa)	Composite (MPa)	Increment (%)	
PALF (20 wt.%)	TRM/CM	33.7	54.6 (57.7)	62 (71)	1256	2567 (2375)	104 (89)	This work
NFM (20 wt.%)	TRM/CM	33.7	39.8 (43.3)	18 (28)	1256	1905 (1607)	52 (28)	This work
Coconut (20 wt.%) *	ITM/CM	19.9	16.5	−17	581	647	11	[37]
Flax (20 wt.%)	TSW/IM	-	-	-	592	636	7	[38]
Maple (30 wt.%) **	Dry blending/CM	19.6	26.2	34	953	1323	39	[56]
Hemp (20 wt.%) ***	IM	22.6	22.6	0	-	-	-	[62]
Sisal (10 wt.%)	SSE/IM	20.3	25.4	25	714	1123	57	[63]
Abaca (30 wt.%)	ITM/IM	21.3	30.1 (50.3)	41 (136)	-	-	-	[64]

Mixing: TRM: Two-roll mill; ITM: Internal mixer; TSE: Twin screw extruder; SSE: Single screw extruder; Molding: CM: Compression molding; IM: Injection molding; * Coconut fiber was treated with 2 wt.% NaOH for 24 h. ** Maple fiber was treated with 1 wt.% MAPE at 90 °C for 30 min. *** Hemp fiber was treated with 5 wt.% NaOH at 50 °C for 24 h.

## Data Availability

The data presented in this study are available on request from the corresponding author.
